# Resonant Vibrational Enhancement of Downhill Energy Transfer in the *C*-Phycocyanin Chromophore Dimer

**DOI:** 10.1021/acs.jpclett.4c02386

**Published:** 2024-11-11

**Authors:** Siddhartha Sohoni, Ping-Jui Eric Wu, Qijie Shen, Lawson T. Lloyd, Craig MacGregor-Chatwin, Andrew Hitchcock, Gregory S. Engel

**Affiliations:** † Department of Chemistry, James Franck Institute, The Institute of Biophysical Dynamics, Pritzker School of Molecular Engineering, The University of Chicago, Chicago, Illinois 60637, United States; ‡ School of Biosciences, University of Sheffield, Sheffield S10 2TN, United Kingdom

## Abstract

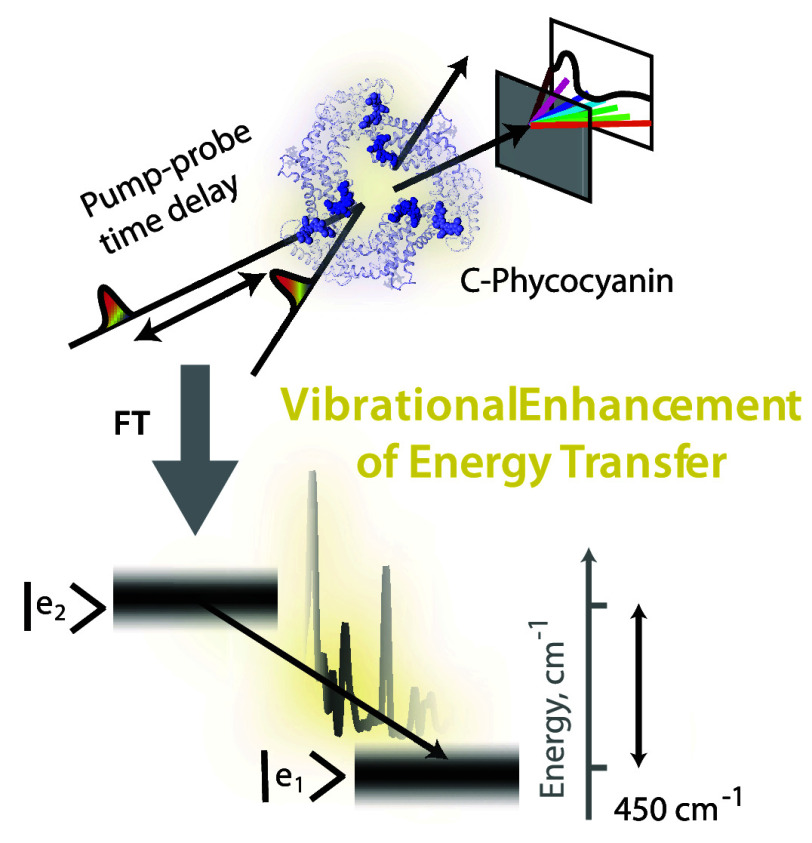

Energy transfer between electronically coupled photosynthetic light-harvesting antenna pigments is frequently assisted by protein and chromophore nuclear motion. This energy transfer mechanism usually occurs in the weak or intermediate system-bath coupling regime. Redfield theory is frequently used to describe the energy transfer in this regime. Spectral densities describe vibronic coupling in visible transitions of the chromophores and govern energy transfer in the Redfield mechanism. In this work, we perform finely sampled broadband pump–probe spectroscopy on the phycobilisome antenna complex with sub-10-fs pump and probe pulses. The spectral density obtained by Fourier transforming the pump–probe time-domain signal is used to perform modified Redfield rate calculations to check for vibrational enhancement of energy transfer in a coupled chromophore dimer in the *C*-phycocyanin protein of the phycobilisome antenna. We find two low-frequency vibrations to be in near-resonance with the interexcitonic energy gap and a few-fold enhancement in the interexcitonic energy transfer rate due to these resonances at room temperature. Our observations and calculations explain the fast downhill energy transfer process in *C*-phycocyanin. We also observe high-frequency vibrations involving chromophore–protein residue interactions in the excited state of the phycocyanobilin chromophore. We suggest that these vibrations lock the chromophore nuclear configuration of the excited state and prevent the energetic relaxation that blocks energy transfer.

Energy transfers from antenna pigments to reaction centers in photosynthesis have near-unity quantum efficiency.^1−3^ The photosynthetic antenna pigment network for electronic excitation flow is coupled to the protein and chromophore nuclear bath. Therefore, energy capture or downhill energy transfer occurs through bath-mediated processes in photosynthesis. Over large distances, energy transfers primarily through a cascade of dipole–dipole interactions between pigments.^4−6^ This process is called Förster resonance energy transfer (FRET). However, when chromophores are separated by subnanometer distances and are electronically coupled, nuclear motion can assist in fast energy transfer between chromophores. This mechanism is referred to as the Redfield-type mechanism,^7−11^ and it provides significant enhancement to the net energy transfer rate.^12−15^

FRET rates are calculated with relative ease when protein cryo-EM structures are known.^16−18^ They require knowledge of dipole orientations, interchromophore distance, absorption and emission spectra, and dipole moments of the donor and acceptor chromophores.^19^ In comparison, the assistance provided by bath vibrations to energy transfer is not calculated in a straightforward manner because the low-frequency (0–800 cm^–1^) vibrations coupling to the energy transfer process are not easily characterized through ultrafast Raman,^20^ resonance Raman,^21^ or ultrafast IR spectroscopy.^22,23^ Typically, broad ohmic spectral densities are used as bath spectral densities that couple to the transfer process.^24−28^ While an ohmic spectral density can statistically describe the system–bath interaction, it does not account for the role of particular vibrations^12,24,26^ that are in near-resonance^29,30^ with the interexcitonic energy gap between the donor and acceptor chromophores. Near-resonance selectively spikes the interexcitonic energy-transfer rate^9,29^ and is, therefore, an important consideration for the rate calculation.

In this work, we explore the earliest energy transfer processes in the phycobilisome light-harvesting antenna of cyanobacteria.^4,18,31^ The phycobilisome antenna utilizes phycocyanobilin as its light-absorbing chromophore (Figure 1a). Unlike free chlorophyll, free phycocyanobilin in solution is floppy and, as a result, highly spectrally tunable, depending on the protein scaffold.^32,33^ π-conjugation in phycocyanobilin depends on the chromophore configuration, and increasing conjugation red-shifts the absorption and emission wavelengths. The antenna is composed of rods of *C*-phycocyanin (CPC) trimers (shown in blue in Figure 1b) and a lateral core assembly of allophycocyanin (APC) trimers (shown in red in Figure 1b). Spectral tunability provides the phycobilisome antenna with a broad absorption feature at 600–650 nm and a fluorescence feature at ∼664 nm (Figure 1c). Within each CPC/APC trimer, two phycocyanobilin chromophores, covalently linked to the α_84_ and β_84_ sites, are within 2 nm of each other^18^ and are, therefore, electronically coupled (Figures 1d and 1e). The β_84_ chromophore has a lower energy than α_84_ due to a more planar chromophore structure in the β pocket. CPC absorbs maximally at 620 nm and emits at 645 nm. APC absorbs at ∼650 nm and emits at 660 nm.^34^ Pump–probe spectra of the phycobilisome antenna have been reported numerous times^34,35^ and feature a prominent excited-state absorption (ESA) feature between 660 and 680 nm.^4^

**Figure 1:**
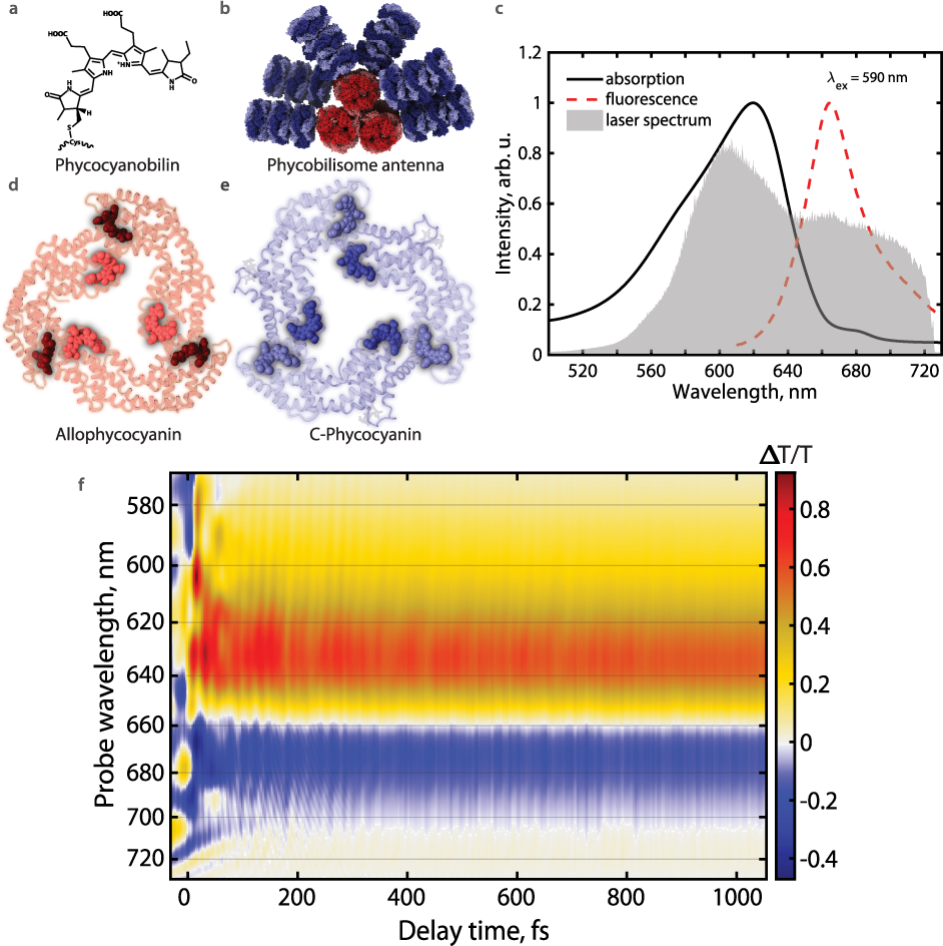
(a) Chemical structure of the phycocyanobilin chromophore. (b) Cryogenic electron microscopy (cryo-EM) structure^18^ of the *Synechocystis* 6803 phycobilisome antenna with *C*-phycocyanin rods colored blue and allophycocyanin cores colored red. (c) Absorption and emission spectra of *Synechocystis* 6803 phycobilisome antenna (adapted from ref (4)). The laser spectrum used for experiments in this work is shown in gray. (d) Allophycocyanin trimer protein structure with the coupled chromophore dimer highlighted. (e) *C*-phycocyanin trimer protein structure with the coupled chromophore dimer highlighted. (f) Transient transmission spectrum of the *Synechocystis* 6803 phycobilisome antenna up to 1.1 ps collected in 3 fs time steps. Negative signal is excited-state absorption; positive signal is ground-state bleach and stimulated emission.

To study energy transfer within the dimer of coupled chromophores, we obtain the profile of the low-frequency spectral density of the nuclear modes coupling to Franck–Condon excitation from pump-probe data for use in modified Redfield theory calculations. Specifically, we perform finely sampled broadband pump–probe spectroscopy on the intact phycobilisome antenna complex with sub-10-fs pulses (Figure 1f). We Fourier transform the obtained time-domain data for each point on the probe wavelength axis. Normalized frequency domain data for multiple probe wavelengths are plotted in Figure 2. The Fourier-transformed data matches within our spectral resolution with resonance Raman experiments on phycocyanobilin-based systems in the high-frequency regime.^20,21,36−39^ Notably, this method allows Rayleigh scatter-free characterization of the low-frequency vibrational modes that are Franck–Condon active^40^ and, hence, couple to electronic excitation. We fit the obtained peaks with generalized Brownian oscillator functions^41^ and use the fit functional form as the spectral density in numerical simulations of modified Redfield theory calculations of interexcitonic energy transfer in the excitonically coupled dimer of CPC.

**Figure 2:**
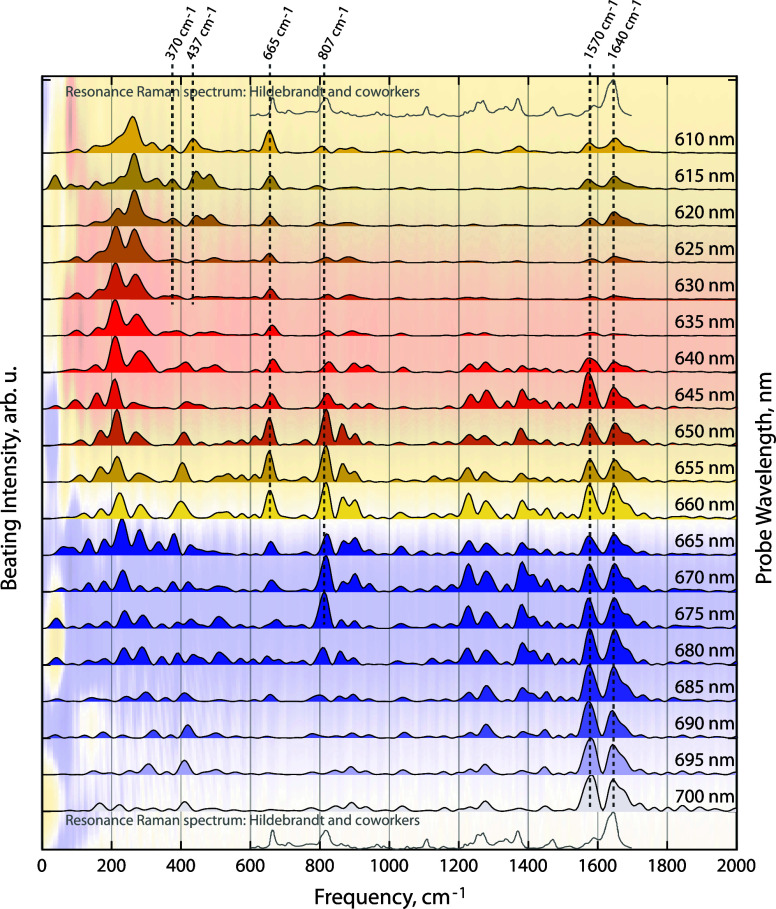
Normalized Fourier transforms of time traces of the phycobilisome pump–probe signal at different wavelengths. The *x*-axis shows the vibrational beating frequency. Intensity is normalized to a maximum at each wavelength. Signal-to-noise is reported in Figures S1 and S2. The resonance Raman spectrum of *C*-phycocyanin from the work of Hildebrandt and co-workers^36^ (digitized with WebPlotDigitizer)^45^ is shown for comparison. Vibrational modes at 370, 437, 665, 807, 1570, and 1640 cm^–1^ are marked with dashed lines.

We find two vibrational modes, at 370 and 437 cm^–1^, to be in resonance with reported literature values of the *C*-phycocyanin interexcitonic energy gap.^27,42^ Modified Redfield theory calculations show a many-fold enhancement in the interexcitonic energy transfer rate due to the involvement of these modes in the transfer process. Our approach provides a broadly applicable method to incorporate realistic vibrational spectral densities into energy transfer rate calculations. Separately, the ESA feature^4^ in phycobilisomes allows us to selectively observe active excited-state vibrational modes that lock the chromophore configuration and prevent energetic relaxation to promote energy transfer.^43^ Specifically, we observe that chromophore-residue hydrogen bonding prevents planarization and energetic relaxation of the phycocyanobilin excited state so that transfer precedes trapping.^44^ Our characterization of the vibrations coupled to the excitons in the phycobilisome antenna highlights the role of both tertiary and primary protein structures in regulating and promoting the energy capture process in oxygenic photosynthesis.

The obtained beating pattern shown in Figure 2 provides information similar to two-dimensional (2D) electronic-vibrational spectroscopy^30,46,47^ but with vibrational resolution only along the visible probe axis. For example, low-frequency modes change systematically across 630–660 nm, clearly indicating two different species in this spectroscopic region, which we know to be *C*-phycocyanin and allophycocyanin, respectively. This information cannot be accessed through steady-state spectroscopy and is not clearly visible in time-domain pump–probe measurements. To calculate vibration-mediated energy transfer rates, we use the Fourier transform of the data at 620 nm, because this wavelength has a low overlap with APC spectral features.

The extracted spectral density is shown in Figure 3a. One can find most of the peaks located in the low-frequency region: the highest intensity peak is at 260 cm^–1^. Given that the Coulomb coupling is −175 cm^–1^, the lowest possible excitonic energy gap of the dimer system is 350 cm^–1^. Therefore, we are particularly interested in the role of the 370 and 437 cm^–1^ peaks in enhancing the downhill energy transfer. The 807 cm^–1^ HOOP mode also appears in the extracted spectral density; however, it has a low relative intensity in comparison to the low-frequency modes. Moreover, the energy gap of the chromophore dimer in CPC is much lower than this frequency, so we do not expect a significant role for the HOOP mode in the excitation energy transfer of the CPC dimer.

**Figure 3:**
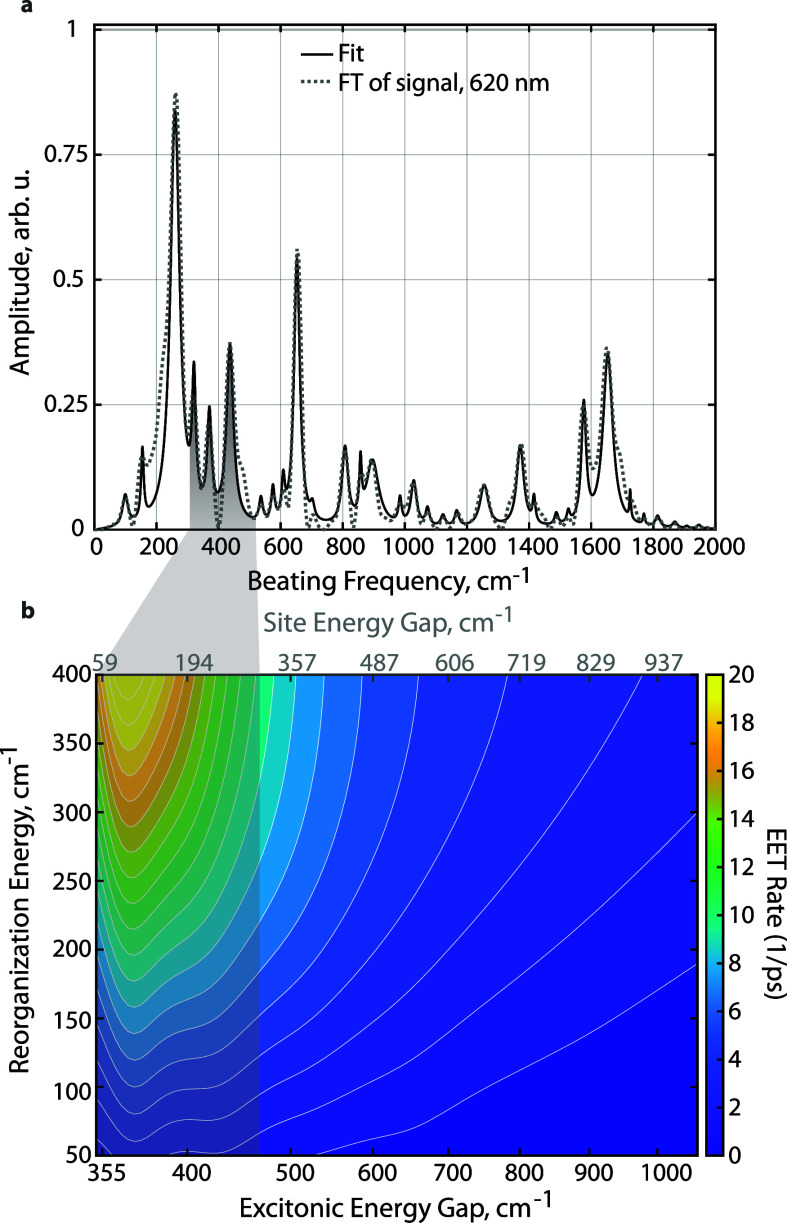
(a) Fourier transform of the probe signal at 620 nm, which we use as the spectral density for *C*-phycocyanin. The Fourier-transformed “vibrational spectrum” is fit with multiple Brownian oscillator peaks. (b) Calculated modified Redfield rates were obtained using the Fourier-transformed spectrum in panel (a) as the spectral density.

Excitation energy transfer in the dimer system is calculated as a function of the reorganization energy of the MBO bath and the site energy gap between two monomers. We plot the rates as a 2D map in Figure 3b. An intense and clear resonant peak shows up at the excitonic energy gap of 370 cm^–1^ and a less-pronounced resonant peak appears at ∼437 cm^–1^. The resonance between vibrations and the electronic energy gap enhances the downhill energy transfer. While commonly used spectral densities such as the Drude–Lorentz form or ohmic form statistically describe the protein environmental effect, they obscure details of the bath mode distribution of the protein. Our approach directly accounts for these details in the spectral density of the vibrations surrounding the donor and acceptor, allowing us to check for vibrational enhancement.

To quantitatively determine the enhanced energy transfer caused by protein bath modes, leaching simulations are performed. We first conducted identical simulations with extracted spectral density excluding the enhancing modes. We observed that the resonant peak disappeared in the leaching simulation (Figure S7), indicating that the observed resonant peaks with full spectral density are indeed due to the 370 or 437 cm^–1^ vibrational modes. Furthermore, we separated the extracted spectral density into high-frequency regions (ℏω > 350 cm^–1^) and low-frequency regions (ℏω < 350 cm^–1^) and performed identical simulations with only the low-frequency region. By subtracting the energy transfer rate calculated with the complete spectral density (Figure 3b) from the energy transfer rate calculated with only low-frequency bath modes (Figure S6a), we obtained the rate difference. Furthermore, we define the enhancement ratio η_enh_ as
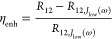
1where *R*_12_ is the modified Redfield rate calculated using completely CPC bath modes, and *R*_12,*J*_low_(ω)_ is the rate calculated using only bath modes with frequency lower than 350 cm^–1^. The result is plotted in Figure S9. Calculated enhancement ratios of up to 84% and 31% for 370 and 437 cm^–1^, respectively, at their resonant peaks when reorganization energy is 200 cm^–1^ emphasize the effect of high-frequency modes. The reported site energy differences between coupled chromophores in CPC are within 70 cm^–1^ to 250 cm^–1^,^42^ shaded gray in Figure 3b. Our results suggest that the CPC protein environment is tuned to facilitate the energy transfer process within the chromophore dimer. This enhancement is the foundation of near-unity energy transfer efficiency in phycobilisomes.

The calculated energy transfer time constants are 91 and 121 fs for site energies of 370 and 430 cm^–1^ with a reorganization energy of 200 cm^–1^, respectively. These time constants are shorter than the experimentally reported time constant (500 fs),^16,27,42,48^ and we attribute this difference to multiple causes. First, the overestimation of rates may imply that the reorganization energy of the system is smaller than 200 cm^–1^. When a reorganization energy of 50 cm^–1^ is used, the time constants for site energies of 370 and 430 cm^–1^ become 361 and 459 fs, which are closer to the experimentally observed time constant of 500 fs but this reorganization energy is probably too small. Measuring the reorganization energy of a monomer within a dimer system is difficult because the emission Stokes shift is affected by the energy transfer process, so the experimental value likely represents upper bounds. Additionally, exciton delocalization complicates the acquisition of monomer properties from a strongly coupled dimer. On the other hand, it may not be merely a matter of reorganization energy; for instance, the extracted spectral density can also dramatically affect the time constant. The spectral density at 620 nm, which corresponds to the CPC absorption maximum, may be contaminated by the signals of vibrational modes of the APC α_84_–β_84_ dimer, which has an absorption maximum at 655 nm. To explore this idea, we performed the same calculation using the spectral density extracted from the 610 nm emission wavelength, which likely has less contamination from APC but risks contamination from β_155_. From the EET rate map in Figure S10, we clearly see that the resonant features remain. And, in Table S2, a comparison between the calculated EET time constants using the spectral density extracted from 620 and 610 nm shows that shifting the spectral density extraction position by 10 nm resulted in 2-fold time constants (196 fs for Δ*E* = 370 cm^–1^, and 193 fs for Δ*E* = 437 cm^–1^ using the reorganization energy of 200 cm^–1^). These time constants are closer to the experimental observed values; however, we cannot conclude that 610 nm is a better wavelength for extracting the spectral density. Although 610 nm is farther from the absorption maximum of APC dimers, the spectral density extracted from 610 nm will have more contamination from the vibrational features of the β_155_ chromophore, which absorbs at absorption is at 600 nm. Lastly, modified Redfield theory could overestimate the energy transfer rates since it assumes state delocalization. For the CPC α_84_–β_84_ dimer system, the scale of the system-bath coupling is the same magnitude as the scales of excitonic coupling and site energy gap. Therefore, dynamic localization occurs, which partially localizes the excitonic states, slowing down the energy transfer process. Even though this process is fast, it will not be complete but the system will rapidly proceed to some pointer state influenced by the bath. Additionally, due to the small site energy gap of the CPC α_84_–β_84_ dimer system and the rapid transfer time, the Markovian approximation can also lead to an overestimation of rate constants. The hierarchical equation of motion (HEOM) approach can address these issues but would require implementing a HEOM with a realistic spectral density. All of the factors mentioned above could lead to the overestimation of rates; nonetheless, none of them affect the resonant features that we observed in this work. Therefore, although modified Redfield theory does not provide accurate quantitative rates, we postulate that it does accurately capture the role of vibrational modes in energy transfer.

Our sub-10-fs-resolved pump–probe spectra allow us to resolve high-frequency vibrations reporting on hydrogen-bonding and conjugation along with the low-frequency vibrations discussed earlier. The probe wavelength dependence of these vibrational peaks can report on the structure and configuration of the chromophore in various protein environments. Our previous work has shown that the prominent negative feature observed in the time-resolved spectra of the phycobilisome complex^31,35,49^ arises from an ESA process in the phycocyanobilin chromophore.^4^ When we Fourier-transform the time domain signal at the ESA wavelengths, we can selectively observe the vibrations that couple to the excited state of the phycocyanobilin, as it is placed in various cavities in the phycobilisome antenna. We observe that two high-frequency modes, at ∼1580 cm^–1^ and ∼1640 cm^–1^, are strongly active on the excited state (Figure 2), implying that the chromophore remains bound along these vibrational coordinates in the excited state. These phycocyanobilin modes are well-characterized in many previous studies, in which they were observed through resonance Raman,^36−39^ time-resolved Raman,^20^ and two-dimensional infrared spectroscopy.^22,50^ Both CPC and APC show this negative ESA feature in their time-resolved spectra. Therefore, it is not possible to assign these vibrations selectively to the APC or CPC excited states. Mutated structures are required for specific studies. However, these modes are observed on the ground electronic state of both^51^ APC and CPC chromophores and important conclusions about protein–cofactor interactions can still be drawn.

Recently, Schlau-Cohen and co-workers^43^ demonstrated, with single-molecule pump–probe spectroscopy, experiments on APC trimers that energy transfer precedes chromophore relaxation in the phycobilisome antenna. This finding is important because the floppy phycocyanobilin molecule relaxes to a highly red-shifted state in free solution.^39^ It shows that the protein environment actively slows this relaxation process. Our selective observation of the excited-state vibrational modes allows us to identify the protein–chromophore interactions that prevent chromophore relaxation in the protein pocket.

The 1580 cm^–1^ mode has been assigned to N–H in-plane modes of the inner rings that hydrogen-bond with the Asp87 residue.^36^ Our density functional theory (DFT)^52^ calculations on the phycocyanobilin with the Asp87 residue in vacuum replicate this assignment. (See Figure S3.^53^) This mode has also been shown to disappear when the chromophore’s inner rings are deprotonated.^37^ The mode at 1642 cm^–1^ is assigned to backbone methine bridge stretching. These modes are shown in Figure 4. pH-dependent studies on the chromophore have shown that relaxation to the so-called far-red trap state in the phycobilin chromophore requires an intermediate in which the chromophore is deprotonated.^38,44^ From this information, we infer that the relaxation of the excited-state landscape to a red emission is prevented by the Asp87 residue holding the inner rings of the chromophore in place through hydrogen bonding, thereby preventing the nuclear motion of the methine bridges to a more conjugated and relaxed configuration. In other words, these modes are strongly active on the excited state with minimal anharmonic shift because the excited-state potential is bound along these coordinates, thus preventing rolling to a globally relaxed minimum. Previous work by Hildebrandt and co-workers^37,54,55^ has shown conclusively that a transient proton release by the phycocyanobilin chromophore in phytochromes precedes photoisomerization. Our observations suggest that the excitation is not coupled with significant proton release, thereby holding the configuration of the chromophore intact. This configuration locking prevents the chromophore from entering a highly conjugated, planar, relaxed state that emits redder than chlorophyll absorption. The locking therefore prevents the formation of traps along the photosynthetic pathway.

**Figure 4:**
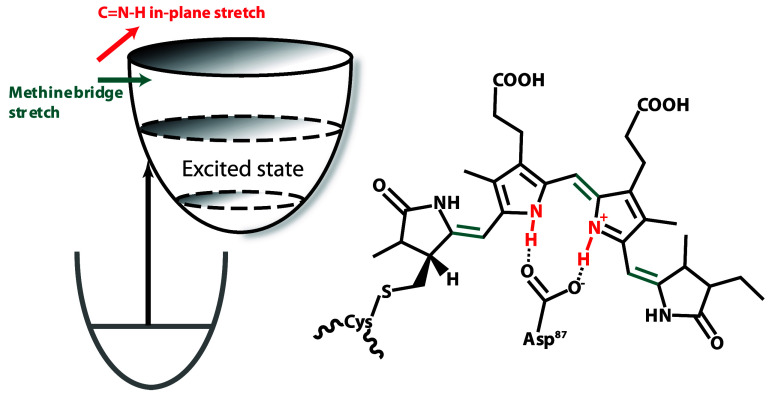
Excited-state potential energy surface remains bound along the 1570 and 1640 cm^–1^ modes corresponding to methine bridge stretching and C=N–H stretch modes. The Asp87 residue that hydrogen bonds with rings B and C of the phycocyanobilin chromophore prevents deprotonation and chromophore relaxation.

Previous works by Moran and co-workers^27,42^ and by Beck and co-workers^56^ have shown that excitons in *C*-phycocyanin are localized on the α_84_ and β_84_ sites and energy transfer between them is not mediated by vibronic coherence. However, we show that, when an excitation is created on the α_84_ site, its transfer to the β_84_ site could still be enhanced through vibrational resonance at room temperature by the Redfield mechanism.^12,13,29^

In this work, we have performed finely sampled broadband pump–probe spectroscopy on the intact phycobilisome complex with sub-10-fs pulses, up to 1.1 ps, before any meaningful long-range energy transfer has occurred in the complex. These experiments were performed to obtain wavelength-dependent spectral densities of the chromophores in the complex. From the wavelength-dependent spectral densities, we made multiple observations about the excited-state dynamics of the chromophores in the phycobilisome antenna. First, we observe that the low-frequency modes of *C*-phycocyanin and allophycocyanin are different in intact phycobilisome. We observe that the 807 cm^–1^ HOOP mode is more active in the allophycocyanin protein than in the *C*-phycocyanin protein, as is the 665 cm^–1^ N–H in-plane mode. Next, we observe that two vibrational modes, at 370 and 437 cm^–1^, couple strongly to the *C*-phycocyanin electronic transitions based on their high cross sections in our time-domain resonance Raman-like spectroscopy. These modes are near-resonant with known excitonic energy gaps for the *C*-phycocyanin chromophore dimer. We utilized the obtained spectral densities in modified Redfield theory calculations. Our calculations show that the presence of these modes enhances the energy transfer by 84%. More generally, our adoption of specific spectral densities in modified Redfield calculations demonstrates a route to incorporate specific nuclear environments into energy-transfer calculations. Lastly, phycocyanobilin has a prominent excited-state absorption feature that allows us to obtain vibrational spectra of the excited states. These spectra reveal that two high-frequency vibrations are active in the excited state. These vibrations involve hydrogen bonding with the protein environment. Hydrogen bonding prevents nuclear planarization of the floppy phycocyanobilin chromophore into a relaxed state, thereby preventing the formation of a trap state. It is likely that this hydrogen bonding is in place to directly favor energy transfer in its competition with relaxation. Our spectra and calculations reveal the multiple chromophore–protein interactions that operate on the single-chromophore level to promote near-unity efficiency energy transfer in this light-harvesting antenna.

## Experimental Methods

### Broadband Pump–Probe Spectroscopy

Phycobilisome isolation and steady-state characterization for this work are reported elsewhere.^4^ Sub-40-fs laser pulses centered at ∼800 nm with an average power of 2.7 W and a repetition rate of 5 kHz are generated in a Ti:sapphire Coherent Legend Elite regenerative amplifier seeded by a Coherent Micra Ti:sapphire oscillator. The laser beam is focused in argon gas at 18 psi. The resulting white-light supercontinuum is compressed to sub-10 fs by using a combination of two DCM10 chirped mirror pairs from Laser Quantum. A representative laser spectrum is shown in Figure 1c. The compressed pulse is split into pump and probe beams with a 90/10 beam splitter. The pump beam is passed through a mechanical delay stage (Aerotech) and chopped at 2.5 kHz (Newport Corp.). The pump and probe are focused into a 200-μm-thick sample cell. The sample is flowed through data acquisition to minimize photodamage. The spot size at the sample cell is ∼250 μm, and the fluence is kept at ∼15 nJ per pump pulse, using reflective neutral density filters. Annihilation is not a concern because it occurs largely beyond the 1 ps delay time of our measurements.^34^ Pump and probe polarizations are kept identical. The probe passes through an iris and is aligned onto a Shamrock spectrometer with a Teledyne Dalsa Spyder 3 CCD camera. Nineteen averages are performed to achieve a high signal-to-noise ratio (Figure S2). Spectra are windowed in detection time (inverse of detection wavelength) to remove pump–probe scatter. Frequency domain data are obtained by Fourier-transforming the obtained time-domain data across the probe wavelength axis after 6-fold zero-padding. Zero-padding is performed to aid curve fitting with Brownian oscillators in MATLAB software. No claims regarding shifts smaller than the original frequency resolution without zero padding are made in the work.

### Curve-Fitting Spectral Densities

To obtain the spectral density *J*(ω) for the rate calculation, we utilized the multimode Brownian oscillator (MBO) spectral density proposed by Meier and Tannor to fit the frequency domain data.^41^
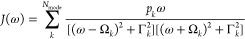
2*N*_mode_ stands for total number of modes, and *p*_*k*_, Ω_*k*_, and Γ_*k*_ are the amplitude coefficient, central frequency, and broadening coefficient of the *k*^th^ mode, respectively. This form of spectral density was originally used to decompose an arbitrary spectral density and provide a numerical solution. Therefore, it is suitable for extracting the spectral density from our experimental beating signal. With a MBO spectral density, frequency domain data at the emission wavelength of 620 nm is chosen to extract the spectral density of the coupled chromophore dimer in CPC. 620 nm is the absorption peak maximum of CPC, and it has low overlap with APC and β_155_ spectral regions. Simulations for 610 nm, with lower APC overlap, are shown in the Supporting Information (SI). The fitting is shown in Figure 3a, and parameters are listed in Table S1. Certain experimental conditions are required for this approach to be accessible. First, the pump and probe pulses should both be sufficiently compressed to observe vibrational frequencies high enough to be of interest. Second, the pulses should not have a positive or negative chirp, because it has been shown that negatively chirped pulses enhance vibrations and positively chirped pulses suppress them.^57^

### Modified Redfield Theory Calculations

To uncover how the protein bath mode enhances energy transfer in the coupled chromophore dimer, we adopted the Frenkel exciton model with the modified Redfield theory in this study. Modified Redfield theory has shown excellent performance in the intermediate region where traditional FRET and Redfield theory fail to capture the correct energy transfer rate, since the system-bath coupling and Coulomb interaction are on the same order of magnitude. Besides, the flexible framework of modified Redfield theory can accommodate various spectral densities without a dramatic increase in computational cost, which allows us to calculate transfer rates with detailed spectral density and obtain a more-realistic energy transfer rate.

The Hamiltonian of coupled chromophore dimer is
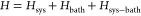
3
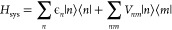
4
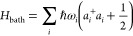
5
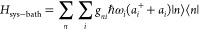
6ϵ_*n*_, *V*_nm_, *ω*_*i*_, *a*_*i*_^+^, α_*i*_, and *g*_*ni*_ are the excitation energy of the *n*th chromophore, Coulomb coupling between the *n*th and *m*th chromophores, the vibrational frequency of the *i*th bath mode, the creation operator of the *i*th bath mode, the annihilation operator of the *i*th bath mode, and the coupling strength of the *i*th bath mode to the *n*th chromophore, respectively. The coupling strength *g*_*ni*_ is a dimensionless quantity used to describe how strongly a vibrational bath mode couples to the electronic state, and it can be related to the mode displacement *d*_*ni*_ by the relation *g*_*ni*_^2^ = *m*_*i*_ω_*i*_*d*_*ni*_^2^/2*ℏ*. Since we are interested in a dimer system, the system Hamiltonian is simply a two-level system.

Modified Redfield theory treats only the off-diagonal system-bath coupling in eigenbasis as perturbation, and it yields the modified Redfield rate from eigenstate β to eigenstate α as
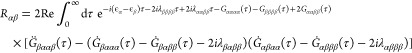
7Greek letters are used as indices of eigenstates, λ is the reorganization energy, and *g*(τ) is the line shape function. Reorganization energy and the line shape function are defined by the spectral density in site basis as
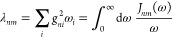
8and
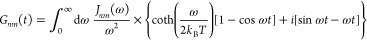
9and, therefore, the reorganization energy and the line shape function in eigenbasis can be expressed as λ_αβγδ_ = ∑_*nm*_*C*_*n*_^α^*C*_*n*_^β^*C*_*m*_^γ^*C*_*m*_^δ^λ_*nm*_ and *G*_αβγδ_(*t*) = ∑_*nm*_*C*_*n*_^α^*C*_*n*_^β^*C*_*m*_^γ^*C*_*m*_^δ^*G*_*nm*_(*t*). With the MBO spectral density proposed by Meier and Tannor, the line shape function and reorganization energy can be calculated analytically.^41^ Additionally, we added an overdamped (OD) spectral density,
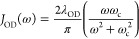
10where *λ*_OD_ and *ω*_c_ are reorganization energy of overdamped spectral density and cutoff frequency, respectively, into the total spectral density to describe the low-frequency bath-mode effect (e.g., ω < 5 cm^–1^) that could not be seen from our broadband pump-probe experiment.

In this study, we calculate the downhill energy transfer rate as a function of the excitation energy difference and reorganization energy of the MBO spectral density to show where the resonant enhancement occurs. Result and discussion are below. The Coulomb coupling is chosen at −175 cm^–1^, the reorganization energy of overdamped spectral density is set at 10 cm^–1^, and the temperature is fixed at 300 K throughout the study.

## Supporting Information Available

Signal-to-noise of spectra, DFT computation details, Redfield simulation details, protein protocols (PDF)
