# Supplementary material for: Resonant Vibrational Enhancement of Downhill Energy Transfer in the C-Phycocyanin Chromophore Dimer
Source: J Phys Chem Lett. 2024 Nov 11;15(46):11569–76. doi: 10.1021/acs.jpclett.4c02386 (PMC11587079; doi:10.1021/acs.jpclett.4c02386)
Supplement: Supplementary file 1 — jz4c02386_si_001.pdf [file jz4c02386_si_001.pdf]

Supplementary Information for

Resonant Vibrational Enhancement of Downhill Energy Transfer in the C-Phycocyanin Chromophore Dimer

Siddhartha Sohoni[1, 3], Ping-Jui Eric Wu[1, 3], Qijie Shen[1,] Lawson T. Lloyd[1], Craig MacGregor-Chatwin[2], Andrew Hitchcock[2], Gregory S. Engel[1]

1. Department of Chemistry, James Franck Institute, The Institute of Biophysical Dynamics, Pritzker School of Molecular Engineering, The University of Chicago, Chicago IL 60637
2. School of Biosciences, University of Sheffield, Sheffield S10 2TN, UK
3. contributed equally

Spectroscopic details

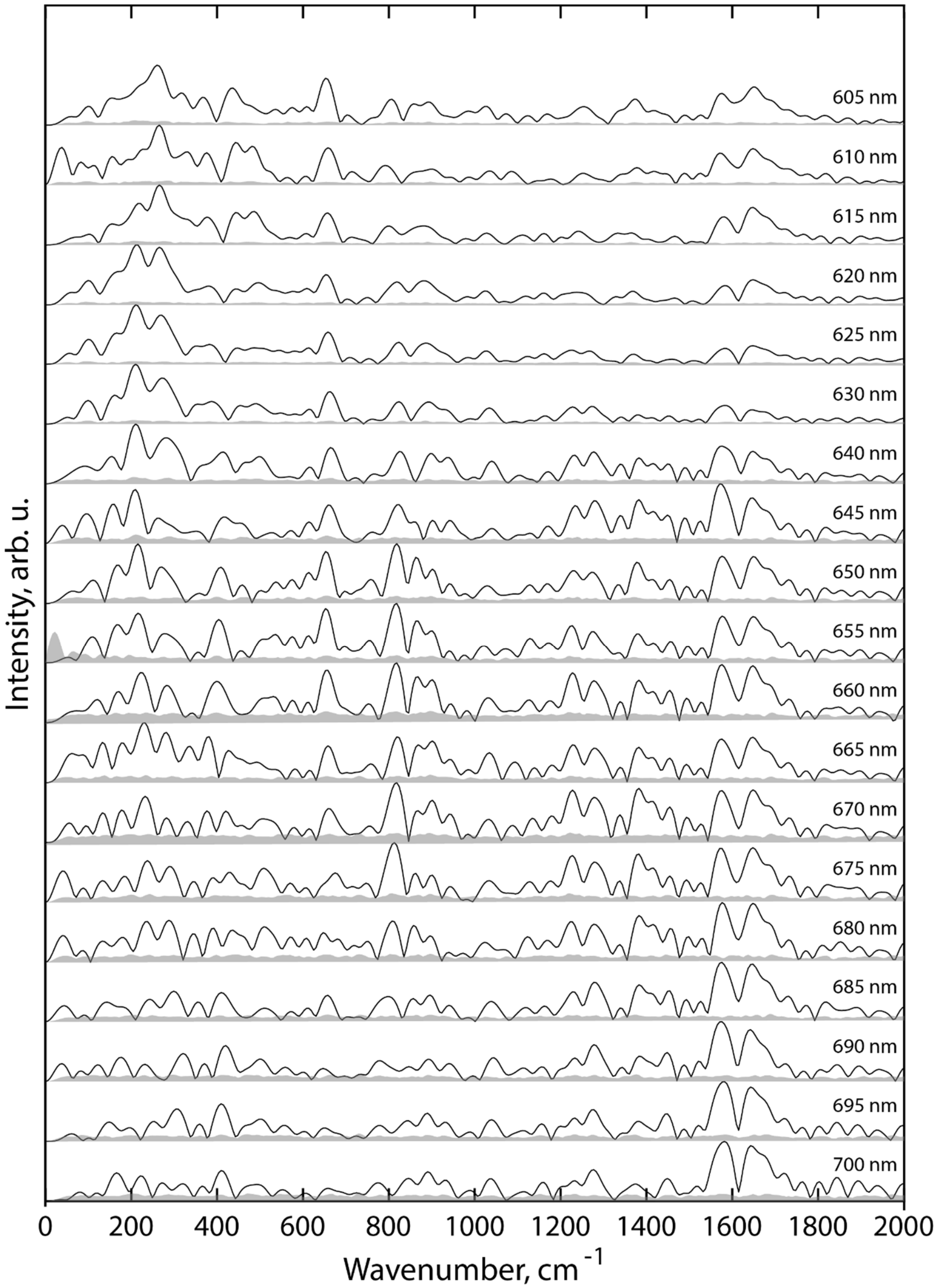

Figure S1: Signal-to-noise ratios for representative wavelengths. Filled curves show the noise floor (standard error) in the frequency domain. Noise is calculated by independently Fourier-transforming individual runs and calculating their combined first and second moments. Solid lines show signal. No claims in the manuscript involve peaks which are lower than the noise floor.

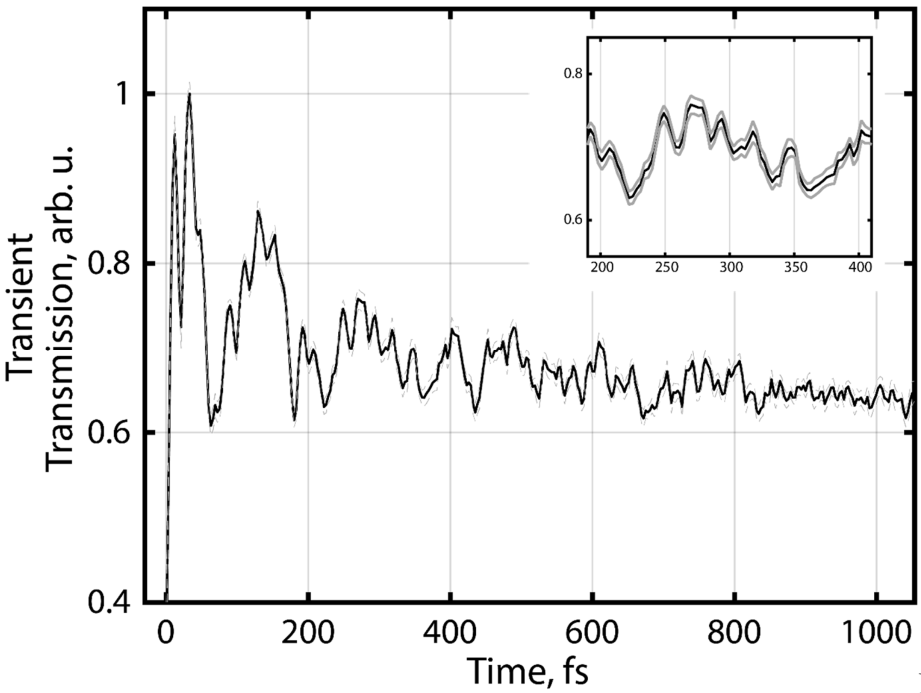

Figure S2: Signal-to-noise in the time domain at 630 nm. Standard error is shown in grey. Averaged signal is shown in black.

Computational Details: Density Functional Theory Calculations

The structure of phycocyanobilin and the amino acid Asp^{87} were extracted from the Protein Data Bank (PDB) file 4F0T (Ref 52, main text). The protonated state of phycocyanobilin was used to study vibrational modes of phycocyanobilin in its natural environment. Density functional theory (DFT) calculations were performed using Gaussian 16 (Revision A.03). The ground state structure of phycocyanobilin was optimized using the B3LYP hybrid functional with the 6-31G(d) basis set in vacuum, with the Asp^{87} residue frozen. Vibrational frequencies were calculated at the same level of theory. The calculations of IR and Raman spectra reported here are all performed on the free chromophore in the ground state. Raman calculations on the excited state with a protein structure have been performed previously by Hildebrandt and coworkers (ref 36, main text) and show that this mode is also preserved on the excited state. We have replicated only the ground state calculations to confirm the identity of the mode.

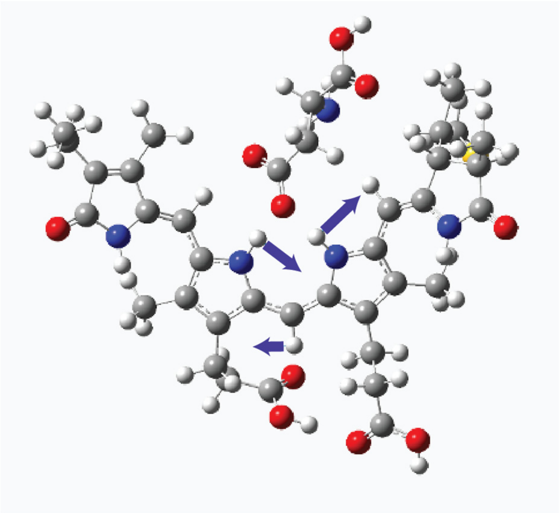

Figure S3: Displacement vector of the IR- and Raman-active 1586 cm^{-1} mode recovered from the DFT calculation shows ring B and ring C in-plane displacement.

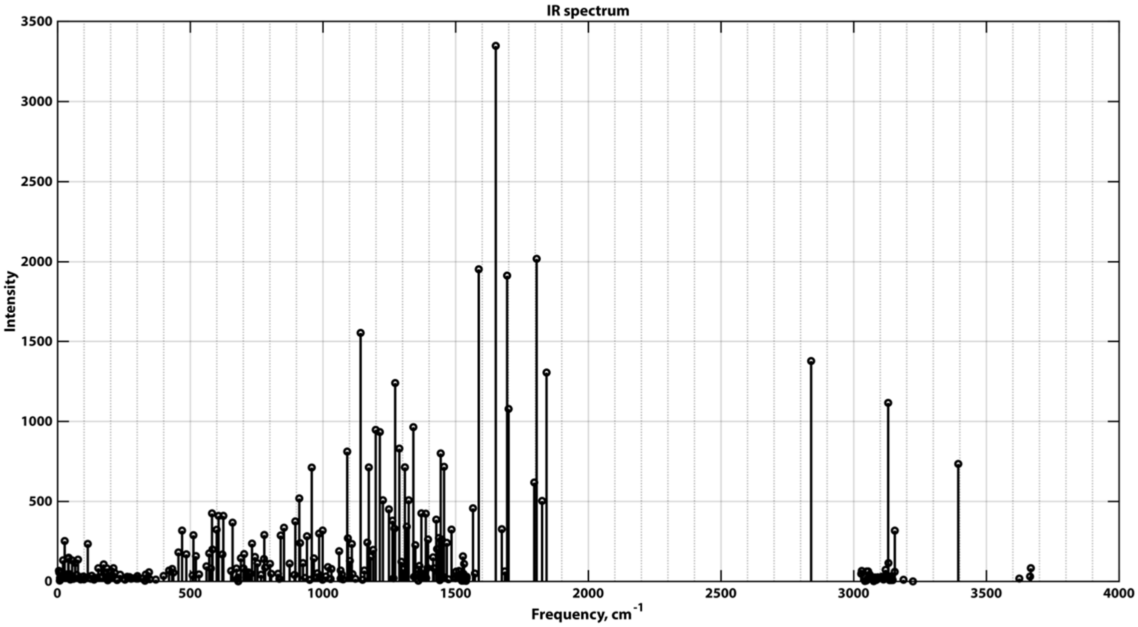

Figure S4: IR spectrum obtained from DFT calculations on the chromophore ground state in vacuum.

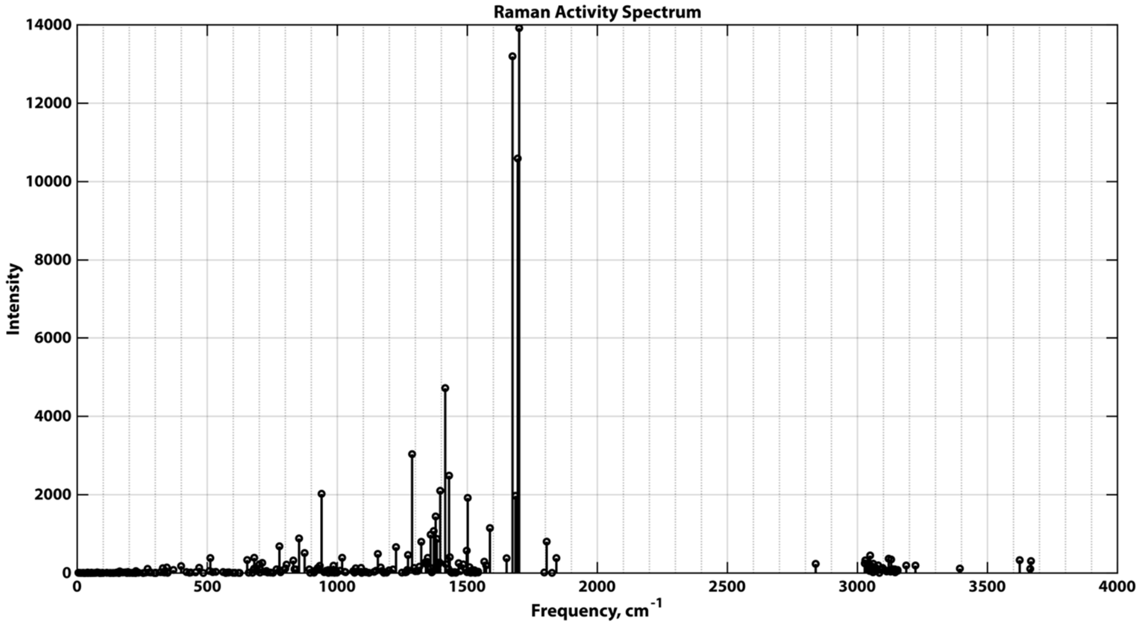

Figure S5: Raman spectrum obtained from DFT calculations performed on the chromophore in vacuum.

Redfield Simulation Details

Table S1: Extracted multimode Brownian oscillator (MBO) parameters of the spectral density of C-Phycocyanin. p_k is normalized to unity and its value will be changed in the rate calculation to tune the reorganization energy of MBO spectral density.

k	p_k	Ω_k, cm^{-1}	Γ_k, cm^{-1}
1	0.0010	99.55	10.12
2	0.0010	154.86	5.11
3	0.1192	259.94	18.07
4	0.0076	320.78	7.38
5	0.0098	370.55	8.73
6	0.0484	436.92	13.57
7	0.0023	536.47	7.15
8	0.0026	575.19	6.24
9	0.0028	608.37	5.96
10	0.0761	652.62	11.38
11	0.0019	702.39	7.45
12	0.0273	807.48	11.45
13	0.0030	857.25	4.42
14	0.1187	895.97	24.30
15	0.0031	984.46	6.01
16	0.0188	1028.70	10.99
17	0.0035	1072.95	7.46
18	0.0033	1122.73	8.66
19	0.0042	1166.97	8.21
20	0.0483	1255.46	16.39
21	0.0721	1371.61	13.80
22	0.0035	1415.85	5.29
23	0.0029	1487.75	6.99
24	0.0021	1526.46	5.83
25	0.0527	1576.24	9.12
26	0.3514	1653.67	19.09
27	0.0012	1725.57	2.72
28	0.0009	1769.81	3.73
29	0.0053	1814.06	8.84
30	0.0028	1869.37	8.61
31	0.0006	1908.08	6.21
32	0.0015	1946.80	7.83
33	0.0001	1974.45	3.63

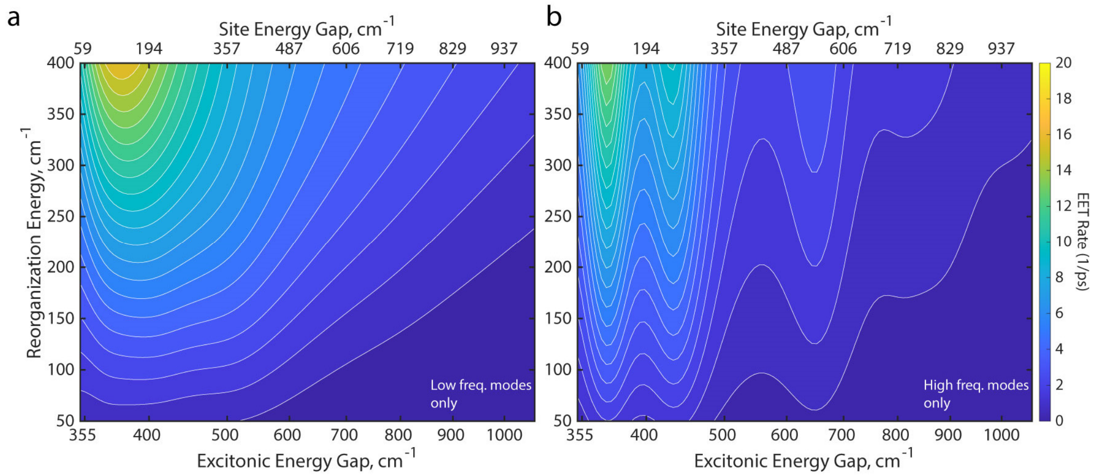

Figure S6: The modified Redfield rates calculated with spectral density having modes with frequencies (**a**) lower than 350 cm^{-1} and (**b**) higher than 350 cm^{-1}.

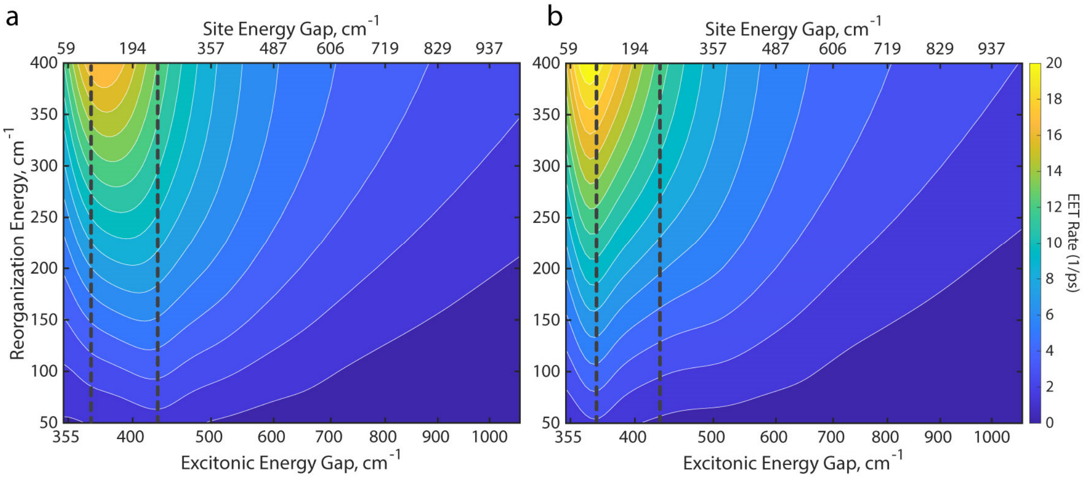

Figure S7: The modified Redfield rates calculated in leaching experiments. (**a**) and (**b**) are the rates calculated from the spectral density excluding the 370.55 cm^{-1} and 436.92 cm^{-1} mode, respectively. The dash lines are located at the excitonic energy gap of 370 and 437 cm^{-1} to emphasize the disappearance of resonant peaks in leaching experiments.

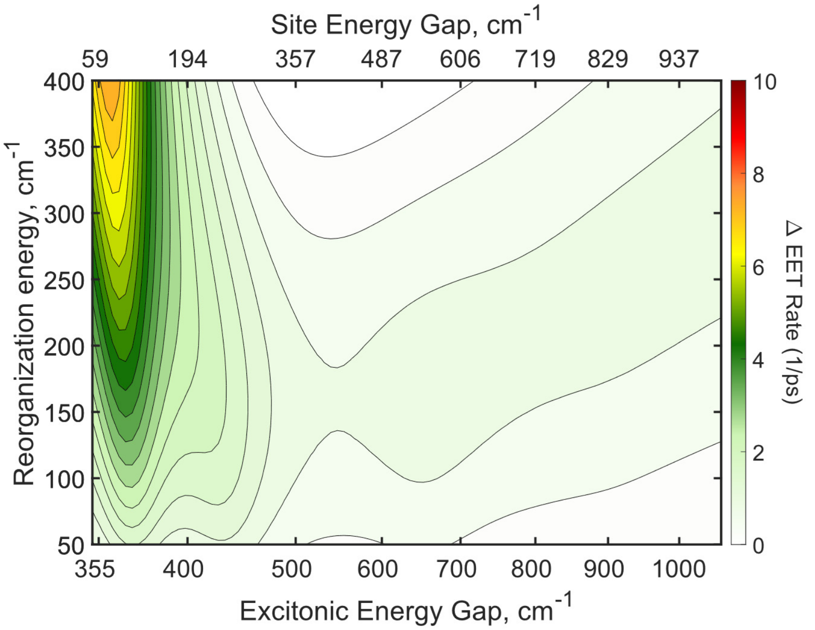

Figure S8: The difference between rates calculated with a complete bath (**Figure 3b**) and rated calculated with only the low frequency modes (**Figure S6a**).

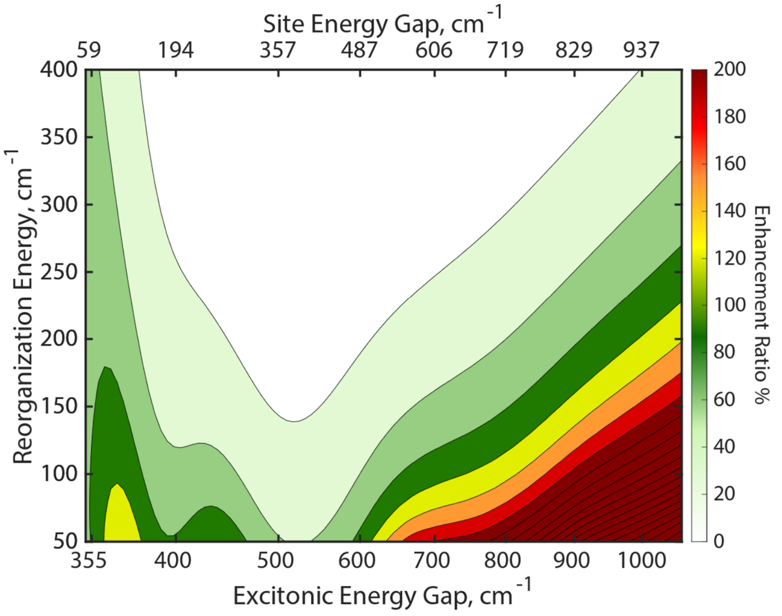

Figure S9: The enhancement ratios of the vibration-assisted energy transfer rate. The enhancement ratios are defined as $\eta_{enh} = \frac{R_{12} - R_{12,J_{low}(\omega)}}{R_{12,J_{low}(\omega)}}$ in the main text.

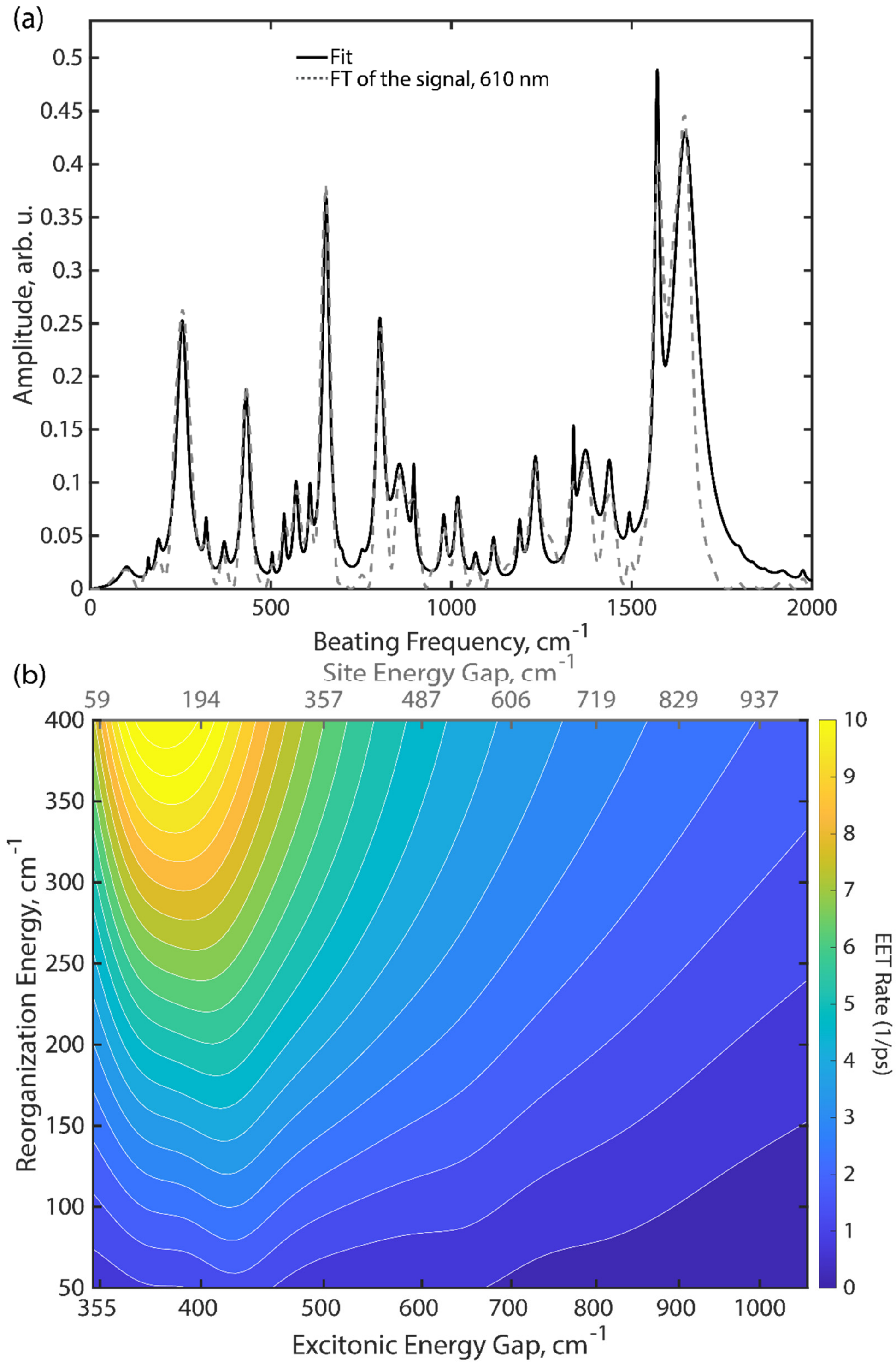

Figure S10: (a) Fourier transform of the probe signal at 610 nm. **(b)** Calculated modified Redfield rates using the Fourier-transformed spectrum in **(a)**.

Table S2: Comparison of the time constants calculated from spectral density extracted from 620 nm and 610 nm. $J_{MBO,620}(\omega)$ and $J_{MBO,610}(\omega)$ refer to the spectral density extracted from the 620 nm and 610 nm emission wavelength from the broadband pump-probe experiment of intact phycobilisomes, respectively.

	τ with $J_{MBO,620}(\omega)$	τ with $J_{MBO,610}(\omega)$
λ = 200 cm^{-1}, ΔE = 370 cm^{-1}	91	196
λ = 200 cm^{-1}, ΔE = 437 cm^{-1}	121	193
λ = 100 cm^{-1}, ΔE = 370 cm^{-1}	184	421
λ = 100 cm^{-1}, ΔE = 437 cm^{-1}	230	360

Unit: fs

Cyanobacterial growth condition details

Cultures of *Synechocystis* sp. PCC6803 were grown in liquid BG-11 medium at 30 °C to an optical density of approximately 0.4-0.6 measured at 780 nm. The light intensity used for growth was approximately 30 μmol of photons m^{-2} s^{-1} and cells were continuously agitated by a magnetic stirrer.

Phycobilisome purification details

Cultures were pelleted by centrifugation at 12000 x g at 4 °C for 30 minutes and subsequently resuspended in buffer containing 25 mM potassium phosphate (pH 7.4), 100 mM NaCl and 10 mM $MgCl_2$; 1000 ml of cell culture was resuspended in approximately 10 ml of resuspension buffer. The resuspended cell suspension was then mixed at a ratio 1:1 with 0.1-mm glass beads and cells were ruptured by 8 rounds of 1 minute of bead beating in a Mini-BeadBeater (BioSpec Products) with cells kept of ice between cycles of bead beating for 3 minutes. The lysed cells and glass beads mixture was then centrifuged at 100000 x g at 4 °C for 4 hours to pellet cell membranes, glass beads and unbroken cells. The supernatant containing the phycobilisomes was then decanted and flash frozen in liquid N_2 before being stored at -80 °C.
